# The Brief Case: *Cardiobacterium hominis* endocarditis in a pediatric patient with congenital heart disease

**DOI:** 10.1128/jcm.01565-23

**Published:** 2024-05-08

**Authors:** James Fisher, Elizabeth M. Garrett

**Affiliations:** 1 Department of Pediatrics, The Pennsylvania State University, Hershey, Pennsylvania, USA; 2 Department of Pathology and Laboratory Medicine, The Pennsylvania State University, Hershey, Pennsylvania, USA; Pattern Bioscience, Austin, Texas, USA

**Keywords:** infective endocarditis, bacteremia, HACEK, blood culture

## CASE

A 15-year-old female presented to a medical center in central Pennsylvania after 3 days of chest pain, headache, fatigue, and fevers (patient reported, Tmax 38.1 C). Her medical history was significant for functional asplenia for which she took prophylactic amoxicillin as well as multiple congenital heart defects including double outlet right ventricle, subpulmonary ventricular septal defect, pulmonary valve atresia, and D-malposition of the great vessels. She had previously undergone pulmonary ductal stenting and the Rastelli procedure with multiple revisions. Her most recent cardiac surgery was 5 years ago. The patient has a previous history of *Staphylococcus aureus* endocarditis 14 years ago and culture-negative prosthetic valve endocarditis 5 years ago. At time of presentation, she lived in a suburban neighborhood with public water, she had no recent travel or dental work, and her only zoonotic exposure was a pet hamster.

On admission, the patient was afebrile (Tmax 37.0 C). Complete blood count (CBC) was normal, but c-reactive protein (CRP; 6.90 mg/dL) and erythrocyte sedimentation rate (ESR; 34 mm/h) were elevated. Respiratory pathogen polymerase chain reaction (PCR) panel and mononucleosis heterophile antibody tests were both negative. Pediatric blood cultures were obtained. No abnormalities were visualized by transthoracic echocardiogram (TTE). By the third day of admission, she was afebrile and had no focal signs of infection and was discharged with outpatient monitoring.

**Fig 1:**
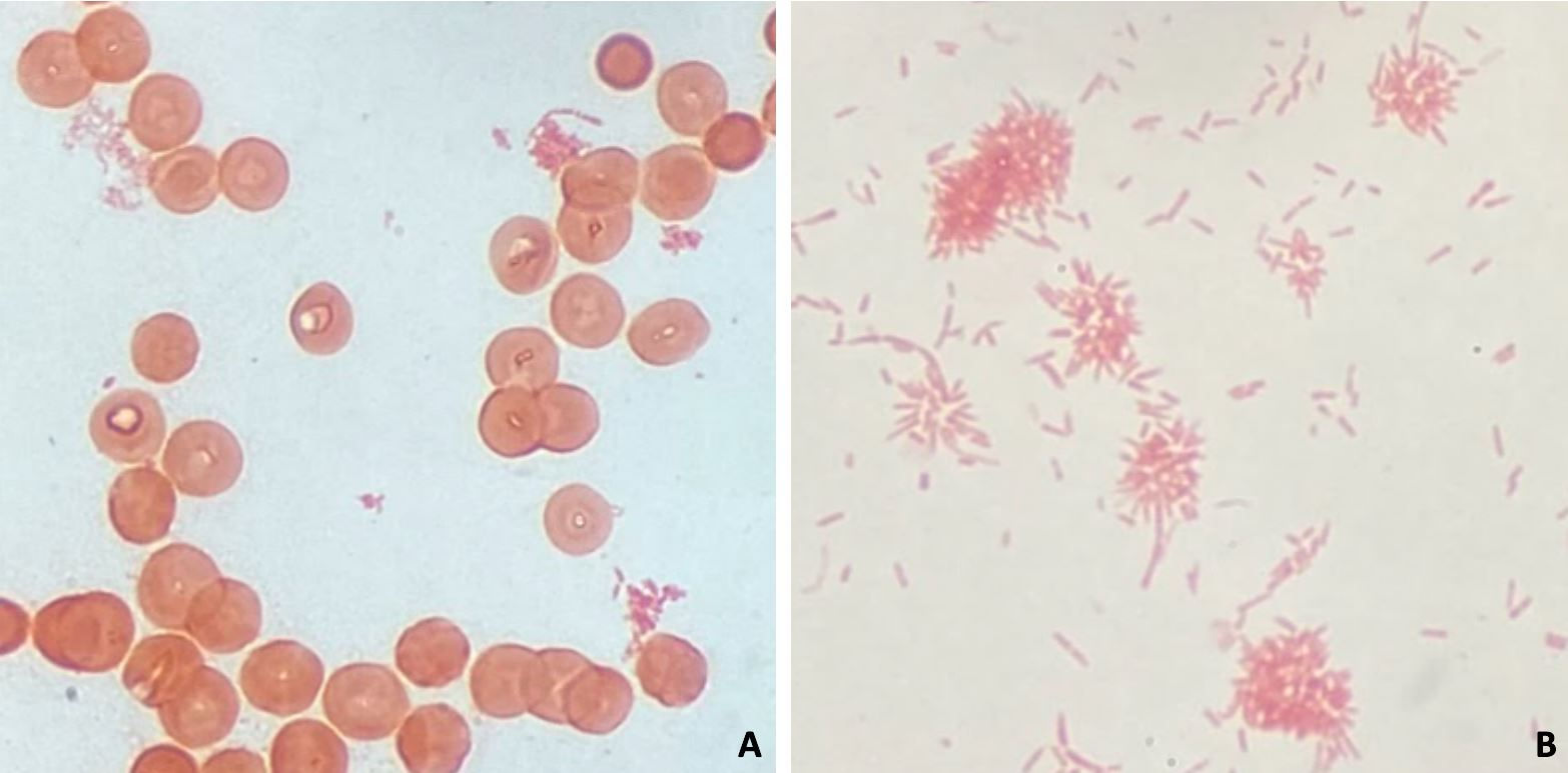
Gram stain of *Cardiobacterium hominis* directly from positive blood culture (**A**) and after growth on blood agar (**B**).

After 89 hours of growth, the aerobic blood culture (BD BACTEC Peds Plus medium) from the first day of admission signaled positive (BACTEC FX). No organisms were seen on Gram stain, and the bottle was incubated for several more hours until again signaling positive. Gram stain revealed faintly staining, small, pleomorphic gram-negative rods (Fig. 1A). The Biofire FilmArray BCID2 panel was negative for all targets. Due to concern for a possible biothreat agent, additional work was performed in a biosafety cabinet. The blood culture was subcultured to blood (tryptic soy agar with 5% sheep’s blood), chocolate, and MacConkey agars. Very faint growth was observed on blood agar after approximately 24 hours of incubation at 35°C with 5% CO_2_. The growth was oxidase and spot indole positive. Initial growth was inadequate for identification by MALDI-TOF mass spectrometry (Bruker sirius) using Bruker MALDI Biotyper CA (v. 3.2.14.8) or research use-only (RUO; v.4.1.80.24) libraries. After 2 days, tiny colonies were apparent on blood agar, with little growth on chocolate and no growth on MacConkey agar. Gram stain showed long, thin gram-negative rods which clumped in rosette-like arrangements (Fig. 1B). MALDI-TOF identification identified the bacterium as *Cardiobacterium hominis* (RUO library; high score 1.89). Subsequent blood cultures collected on days 2 and 3 of hospitalization grew the same organism. Antibiotic sensitivities were unable to be obtained due to poor growth of the organism, and a β-lactamase test was not performed at the time.

Due to the positive blood cultures, the patient was admitted 2 days after initial discharge, still afebrile and feeling overall well. Lab results were consistent with her first admission, with normal CBC and elevated CRP and ESR. She was initially started on empiric ceftriaxone (50 mg/kg twice daily, maximum 2,000 mg), which was continued after identification of *C. hominis*. Blood cultures collected after initiation of ceftriaxone were negative. TTE still did not demonstrate any vegetations, but visualization was noted to be challenging due to the presence hardware. On the basis of her clinical presentation, culture results, history of endocarditis without an organism identified, and predisposing factors, the patient was diagnosed with probable sub-radiographic endocarditis. After 4 days of treatment, the patient developed pseudolithiasis of the gallbladder that was attributed to ceftriaxone. She was subsequently transitioned to cefepime (50 mg/kg every 8 hours, maximum 2,000 mg) and successfully completed a 6-week outpatient course.

## DISCUSSION

Bacteria of the genus *Cardiobacterium* are fastidious gram-negative bacilli from two species, *C. hominis* and *C. valvarum. Cardiobacterium* are normal human oral and upper respiratory flora. Though this organism is rarely a cause of infection, endocarditis is the most common infectious presentation, and *Cardiobacterium* bacteremia is associated with endocarditis in approximately 90% of cases (1). *Cardiobacterium* is within the HACEK group of bacteria that includes *Haemophilus*, *Aggregatibacter*, *Eikenella corrodens*, and *Kingella*. These organisms together account for less than 5% of all infective endocarditis in both adult and pediatric patients but are highly fastidious and, therefore, are commonly suspected in cases of blood culture-negative endocarditis (2–4). Infection is typically presumed to stem from an oral source. Though rare, *Cardiobacterium* has been isolated from extracardiac infections including pneumonia and osteomyelitis (1, 5).

**Fig 2:**
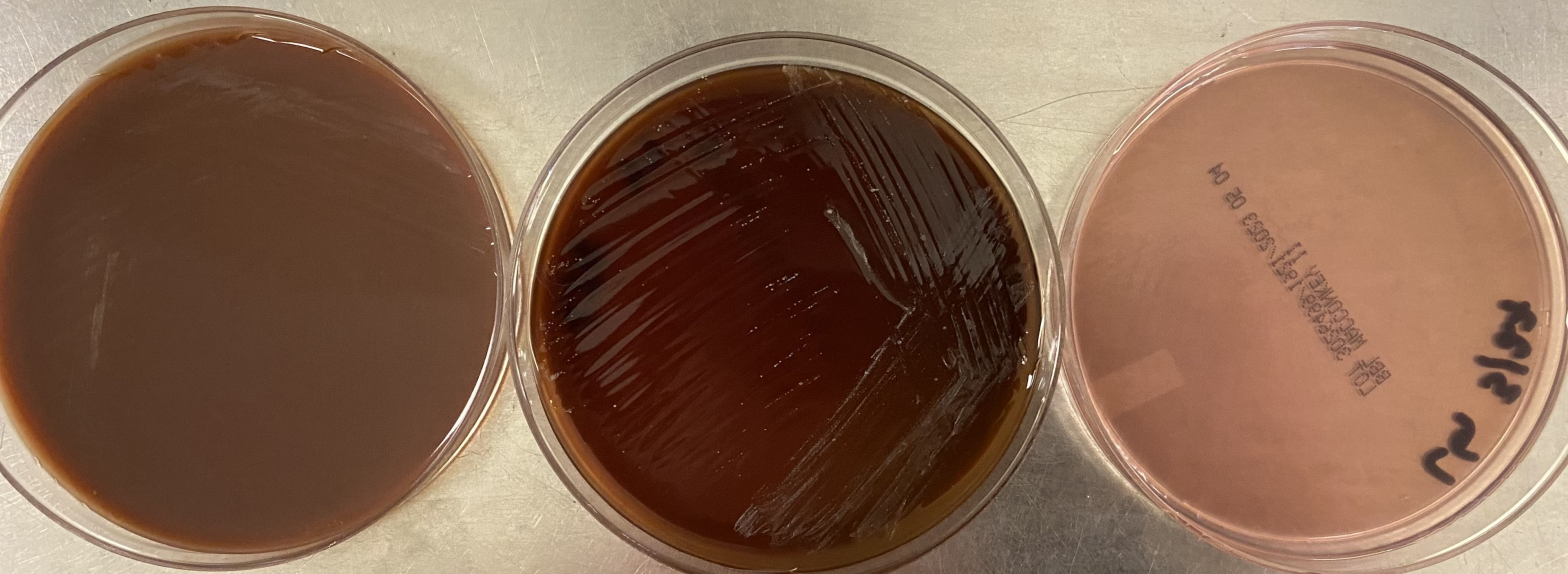
Growth of *C. hominis* on chocolate (left), blood (center), and MacConkey (right) agars after 4 days of incubation at 35°C with 5% CO_2_.

*Cardiobacterium* is a fastidious, slow-growing, and capnophilic bacterium and typically requires at least 48 hours of growth at 35°C with 5% CO_2_ (6). It grows on enriched media including blood and chocolate agar but will not grow on selective media such as MacConkey (Fig. 2). Colonies are typically small, smooth, may be lightly alpha-hemolytic after prolonged growth, and may pit the agar. On Gram stain, *Cardiobacterium* may appear poorly stained and pleomorphic with short coccobacilli as well as long filaments. The bacilli may clump together at one end and form a characteristic “rosette” arrangement as seen in this case. Direct exam from blood culture may not show organisms at all due to the poor ability of this bacterium to retain stain. *Cardiobacterium* species are catalase negative, oxidase, and indole positive, but spot indole may be variable. Identification can be performed by MALDI-TOF mass spectrometry, though it may be challenging to get sufficient growth to do so. Of note, *Cardiobacterium* species are not present in the IVD-marked Bruker CA or VITEK libraries at the time of publication, but *C. hominis* is present in RUO databases. Additional safety measures such as handling cultures in a biosafety cabinet should be considered when encountering unidentified, fastidious, poorly staining gram-negative organisms from blood cultures as there is a risk of exposure to *Francisella tularensis* or similar organisms with biosafety concerns.

*Cardiobacterium* endocarditis, like that caused by other HACEK organisms, typically ha*s* a slow onset with the gradual development of symptoms including chest pain, fatigue, low fever, night sweats, and malaise (2, 7, 8). Subacute symptoms can persist for days to months prior to diagnosis. Pre-existing heart disease and congenital heart defects are risk factors for infection, particularly in pediatric populations (2, 3, 7, 9). *Cardiobacterium* is more commonly associated with infection of the aortic valve and can cause both native and prosthetic heart valve endocarditis (2, 8). Several reports demonstrate infection associated with right ventricle to pulmonary artery conduits in pediatric patients, similarly to this patient (3, 10, 11). In contrast to the case presented, vegetations that may be large and friable are frequently seen; in this case, infection may have been detected before the growth of such vegetations. Stroke is the most common complication; however, HACEK endocarditis has lower mortality compared to that of non-HACEK organisms (<15%) (2, 9). *C. hominis* is more frequently isolated in cases of infection than *C. valvarum*, though it is unclear whether any clinical differences exist between the two species.

Due to their fastidious nature, cases of suspected culture-negative endocarditis are frequently attributed to HACEK organisms including *Cardiobacterium*. In reality, HACEK organisms likely account for a small percentage of all cases of culture-negative endocarditis (2, 12). The predominant causes of blood culture-positive endocarditis (*Staphylococcus*, *Streptococcus*, and *Enterococcus*) are likely underlying most cases of culture-negative endocarditis as well (12, 13). Failure to recover a nonfastidious organism in blood culture may often be attributed to the administration of antibiotics prior to collection. Molecular techniques such as broad range 16S rRNA PCR of biopsies, explanted heart valves, or vegetations can aid diagnosis of fastidious, nonviable, and nonculturable organisms (12, 13). Similarly, next-generation sequencing of microbial cell-free DNA from blood may identify infectious organisms, including HACEKs, even in cases with negative cultures and in patients with prior antibiotic exposure (14, 15). While the current availability of such testing is limited, the application of molecular techniques for diagnosis of these challenging infections is promising.

Historically, the recovery of HACEK organisms, including *Cardiobacterium,* in blood culture was thought to require extended incubation. However, continuous monitoring blood culture systems and modern medium formulations allow the recovery of these fastidious organisms within the standard incubation time of 5 days. While a wide range in the time to detection (2–42 days) has been reported, the median time to recovery is approximately 3–4 days (2, 7). Additionally, due to the low incidence of *Cardiobacterium* and other HACEK organisms in blood cultures, clinical laboratories may find little utility in preemptively holding blood cultures for extended periods. Several studies have found through prospective trials that incubation of blood cultures greater than 7 days does not increase the recovery of HACEKs, though may result in more contaminants (16, 17). Consultation between providers and a clinical laboratory director in cases where an atypical cause of infective endocarditis is suspected can be useful to assess the appropriateness of additional culture methods.

Antibiotic susceptibility testing can be performed for HACEK organisms including *Cardiobacterium* by broth microdilution as described in the Clinical and Laboratory Standards Institute M45 (18). However, sufficient growth may be difficult to achieve. *Cardiobacterium* isolates are predominantly susceptible to β-lactams, including ceftriaxone as well as fluoroquinolones. Rare resistance to penicillin and ampicillin due to β-lactamase production has been reported, so a β-lactamase test such as the chromogenic cephalosporin method may be performed to assess susceptibility to penicillins (19). Ceftriaxone, ciprofloxacin, or ampicillin (for β-lactamase-negative isolates) for 4 (for native valve) to 6 (for prosthetic valve) weeks is currently recommended for treatment (19). Approximately half of the patients require surgical management as well for the removal of vegetations and infected valves (7).

In summary, clinical microbiology laboratories should be suspicious of atypical organisms such as *Cardiobacterium* when a blood culture yields poorly staining, pleomorphic gram-negative rods after 2–4 days of growth. “Rosette” arrangements of gram-negative rods are highly suggesting of *Cardiobacterium* as well. Recovery of this organism in a blood culture should lead to a presumptive diagnosis of infective endocarditis, even if the patient is only experiencing subacute, nonspecific symptoms such as fever and fatigue. While recovery and growth of this organism are challenging, it can be achieved using standard blood culture procedures and media.

## SELF-ASSESSMENT QUESTIONS

1. *Cardiobacterium* is a bacterium that is common in which type of reservoir?
  - Human oral flora
  - Human gastrointestinal flora
  - Rodents such as hamsters
  - Cat feces
2. Which statement is correct regarding *Cardiobacterium* culture?
  - *C. hominis* requires anaerobic growth.
  - *C. hominis* can be recovered using gram-negative selective media such as MacConkey agar.
  - *C. hominis* is unable to grow in standard blood culture media.
  - *C. hominis* is best recovered with a CO_2_ -rich aerobic environment.
3. Which of the following antibiotics are recommended for *Cardiobacterium* endocarditis due to frequent susceptibility?
  - Ampicillin
  - Daptomycin
  - Gentamicin plus penicillin
  - Ceftriaxone

## ANSWERS TO SELF-ASSESSMENT QUESTIONS

1. *Cardiobacterium* is a bacterium that is common in which type of reservoir?
  - Human oral flora
  - Human gastrointestinal flora
  - Rodents such as hamsters
  - Cat feces

Answer: a. *Cardiobacterium* as well as other HACEK organisms are components of the human oral microbiota, and infection is typically presumed to stem from an oral source. This organism is not known to be a normal component of human gastrointestinal flora nor is it considered zoonotic.

- Which statement is correct regarding *Cardiobacterium* culture?
  - *C. hominis* requires anaerobic growth.
  - *C. hominis* can be recovered using gram-negative selective media such as MacConkey agar.
  - *C. hominis* is unable to grow in standard blood culture media.
  - *C. hominis* is best recovered with a CO_2_-rich aerobic environment.

Answer: d. *Cardiobacterium* species can be grown with incubation at 35°C with 5%–7% CO_2_. It can grow on blood agar and in standard blood culture media, though growth may take several days. It is inhibited by MacConkey agar.

- Which of the following antibiotics are recommended for *Cardiobacterium* endocarditis due to frequent susceptibility?
  - Ampicillin
  - Daptomycin
  - Gentamicin plus penicillin
  - Ceftriaxone

Answer: d. Ceftriaxone is the preferred therapy for HACEK endocarditis. HACEK organisms including *Cardiobacterium* are frequently β-lactam susceptible but rarely carry β-lactamases that confer resistance to ampicillin, so *in vitro* susceptibility or a negative β-lactamase test is recommended before treatment with ampicillin. Daptomycin is not active against this organism, and there is no evidence for increased efficacy of combination therapy with gentamicin.

**TAKE-HOME POINTS**

- *Cardiobacterium* is a fastidious, gram-negative organism that is a rare cause of infection but, in cases of bacteremia, is nearly always associated with infective endocarditis.
- *Cardiobacterium* endocarditis is typically slow to develop, with the long-term progression of subacute symptoms such as malaise, mild fevers, and chest pain. Congenital heart defects are a significant risk factor for infection.
- HACEK organisms, including *Cardiobacterium,* can be recovered using modern blood culture systems within a standard incubation time of 5 days. HACEK organisms are a rare cause of blood culture-negative endocarditis.
